# Lysosomal ceramide generated by acid sphingomyelinase triggers cytosolic cathepsin B-mediated degradation of X-linked inhibitor of apoptosis protein in natural killer/T lymphoma cell apoptosis

**DOI:** 10.1038/cddis.2015.82

**Published:** 2015-04-09

**Authors:** M Taniguchi, H Ogiso, T Takeuchi, K Kitatani, H Umehara, T Okazaki

**Affiliations:** 1 Department of Life Science, Medical Research Institute, Kanazawa Medical University, 1-1 Daigaku, Uchinada, Ishikawa 920-0293, Japan; 2 Division of Hematology/Immunology, Department of Medicine, Kanazawa Medical University, 1-1 Daigaku Uchinada, Uchinada, Ishikawa 920-0293, Japan; 3 Department of Biobank, Tohoku Medical Megabank Organization, Tohoku University, 1-1 Seiryo, Aoba, Sendai 980-8574, Japan; 4 Department of Rheumatology and Clinical Immunology, Kyoto University, Sakyo-ku, Kyoto 606-8507, Japan

## Abstract

We previously reported that IL-2 deprivation induced acid sphingomyelinase-mediated (ASM-mediated) ceramide elevation and apoptosis in an NK/T lymphoma cell line KHYG-1. However, the molecular mechanism of ASM–ceramide-mediated apoptosis during IL-2 deprivation is poorly understood. Here, we showed that IL-2 deprivation induces caspase-dependent apoptosis characterized by phosphatidylserine externalization, caspase-8, -9, and -3 cleavage, and degradation of X-linked inhibitor of apoptosis protein (XIAP). IL-2 re-supplementation rescued apoptosis via inhibition of XIAP degradation without affecting caspase cleavage. However, IL-2 deprivation induced ceramide elevation via ASM in lysosomes and activated lysosomal cathepsin B (CTSB) but not cathepsin D. A CTSB inhibitor CA-074 Me and knockdown of CTSB inhibited ceramide-mediated XIAP degradation and apoptosis. Inhibition of ceramide accumulation in lysosomes using an ASM inhibitor, desipramine, decreased cytosolic activation of CTSB by inhibiting its transfer into cytosol from the lysosome. Knockdown of ASM also inhibited XIAP degradation and apoptosis. Furthermore, cell permeable *N*-acetyl sphingosine (C_2_-ceramide), which increases mainly endogenous d18:1/16:0 and d18:1/24:1 ceramide-like IL-2 deprivation, induced caspase-dependent apoptosis with XIAP degradation through CTSB. These findings suggest that lysosomal ceramide produced by ASM mediates XIAP degradation by activation of cytosolic CTSB and caspase-dependent apoptosis. The ASM–ceramide–CTSB signaling axis is a novel pathway of ceramide-mediated apoptosis in IL-2-deprived NK/T lymphoma cells.

KHYG-1 natural killer T (NK/T) cells established from a patient with aggressive NK/T cell lymphoma displayed morphology of a large granular lymphocyte with a large nucleus, rough chromatin, and bulk basophilic cytoplasm.^1^ Suck *et al.*^2^ reported that KHYG-1 cells had *in vitro* cytotoxicity against lymphoma cell lines such as HL-60 and induced apoptosis of tumor cells via the granzyme M/perforin pathway. Thus KHYG-1 cells have a cytotoxic ability similar to NK cells against malignant cells. However, because interleukin-2 (IL-2) is essential for clonal expansion of KHYG-1 cells, this cell line is a useful model to investigate the mechanism by which NK/T lymphoma cells undergo programmed cell death. Previously, we reported that IL-2 deprivation (IL-2(−)) promoted ceramide generation due to the activation of acid sphingomyelinase (ASM), resulting in apoptosis of KHYG-1 cells.^3^ The mechanisms of ASM–ceramide-mediated apoptotic signal in IL-2 deprived NK/T lymphoma cells has not been clarified.

Apoptosis uses two major signaling pathways to induce programmed cell death; extrinsic and intrinsic pathways.^4^ The extrinsic pathway is mediated by extracellular death ligands (Fas ligand, tumor necrosis factor-*α* (TNF-*α*), TNF-related apoptosis-inducing ligand (TRAIL), or CD95) and activates death-inducing signaling complex-containing caspase-8 through those receptors. The intrinsic pathway mediates the disruption of mitochondria and induced the formation of an apoptosome complex composed of cytochrome *C* secreted from mitochondria, apoptotic protease-activating factor 1 (Apaf-1), and caspase-9.^4^ Both extrinsic and intrinsic pathways lead to the activation of caspase-3/-7, which trigger various apoptotic phenomena such as phosphatidylserine (PS) externalization or DNA fragmentation. In addition to these mechanisms that induce apoptosis, it is important to clarify the role of lysosomal proteases in the regulation of antiapoptotic proteins such as Bcl-2 family members and pro-apoptotic proteins such as caspases.

Cathepsins (CTSs) in lysosomes consist of cysteine protease, aspartic protease cathepsin D (CTSD) and serine protease cathepsin B (CTSB). Upon extracellular and intracellular stressors, CTSs are released into the cytosol and are activated enzymatically by optimal pH conditions.^5^ Apoptotic signals mainly inhibit antiapoptotic molecules such as Bcl-2 to activate pro-apoptotic Bax/Bak molecules by their degradation through CTSs.^6, 7^ Currently, the role of CTSD and CTSB in apoptosis is controversial. The activation of CTSB through transforming growth factor-*β* signaling was reported to increase the proliferation of melanoma cells and short hairpin RNA (shRNA) of CTSB had an apoptotic effect mediated through the degradation of X-linked inhibitor of apoptosis protein (XIAP) in invasive meningioma cells, suggesting the positive effect of CTSB in cell proliferation.^8, 9^ In contrast, it was reported that CTSB induced apoptosis by activating caspase-3 and -9 in dengue virus-infected HepG2 hepatocytes.^10^

Caspase-3, -7, and -9 are inhibited by XIAP, an IAP family member^11, 12^ that directly binds to and inactivates caspase-3 or caspase-9 to inhibit their degradation, resulting in suppression of apoptosis.^13, 14, 15, 16^ Downregulation of XIAP increases the sensitivity of cancer cells to apoptotic stimuli, such as TRAIL or hypoxia.^17, 18^ In hematological malignancies, anti-CD33 antibodies induced apoptosis by decreasing XIAP in acute myeloid lymphoma (AML),^19^ and AML patients with overexpression of XIAP showed unfavorable responses to induction chemotherapy.^20^ Anticancer drug-resistant lymphoma cells also had overexpression of XIAP through the NF-*κ*B-dependent MEK/MAPK pathway.^21^ In general, cytosolic proteins such as XIAP are regulated at the transcriptional level or by enzymatic degradation via proteases. However, the molecular mechanism by which the protein levels of XIAP are regulated in ceramide-induced NK/T cell apoptosis has not been investigated.

Ceramide is at the center of sphingolipid metabolism and acts as a substrate of other sphingolipids, such as sphingomyelin, sphingosine-1-phosphate (S1P), and glycosphingolipids.^22, 23^ Ceramide is also a lipid mediator that induces programmed cell death, differentiation, senescence, cell cycle arrest, and autophagy.^22, 23, 24^ There are three pathways in intracellular ceramide generation: (i) *de novo* synthesis from L-serine and a palmitoyl-coenzyme A, (ii) the sphingomyelin cycle consisting of sphingomyelin synthase (SMS) and sphingomyelinase, and (iii) the salvage pathway where ceramide synthases utilize sphingosine degraded from SM, glycolipids, and S1P as a substrate of ceramide.^22^ These pathways are mutually involved in the generation of ceramide induced by various stimuli.^23^ Especially, ASM-generated ceramide has been well investigated in numerous types of cell death. Stimulation of TRAIL or CD95 ligands induces rapid ASM activation and formation of ceramide-enriched platforms in the plasma membrane.^25, 26^ ASM-generated ceramide provides a place to form clusters between ligands and their transmembrane receptors, which transduce an efficient death signal to the intracellular compartment.^25, 26, 27^ However, stimulation of TNF-*α* or gemcitabine generated ceramide in lysosomes through ASM activation. Lysosomal ceramide was reported to trigger the CTSD-mediated apoptotic pathway.^28, 29, 30^ Recently, arsenic trioxide induced the degradation of XIAP through the ubiquitin–proteasome pathway and treatment with valproic acid increased CTSB-induced apoptosis of chronic lymphoid lymphoma cells.^31, 32^

However, how ASM-generated lysosomal ceramide is related to the cathepsin family, including CTSB and CTSD and XIAP in NK/T lymphoma cell apoptosis, is poorly understood. In this study, we demonstrated that IL-2(−) activated the ASM–ceramide pathway in lysosomes and that generation of d18:1/16:0 and d18:1/24:1 lysosomal ceramides caused the release of CTSB, but not CTSD, into the cytosol. CTSB-mediated degradation of XIAP subsequently induced activation of caspase-3 and its nuclear entry to execute apoptosis. These results suggest the ASM/ceramide/CTSB axis is a novel pathway for the degradation of XIAP in IL-2-deprived NK/T lymphoma cell apoptosis.

## Results

### IL-2 rescued cells from caspase-dependent apoptosis without inhibition of caspase-3 cleavage in IL-2(−) condition

**Figure 1:**
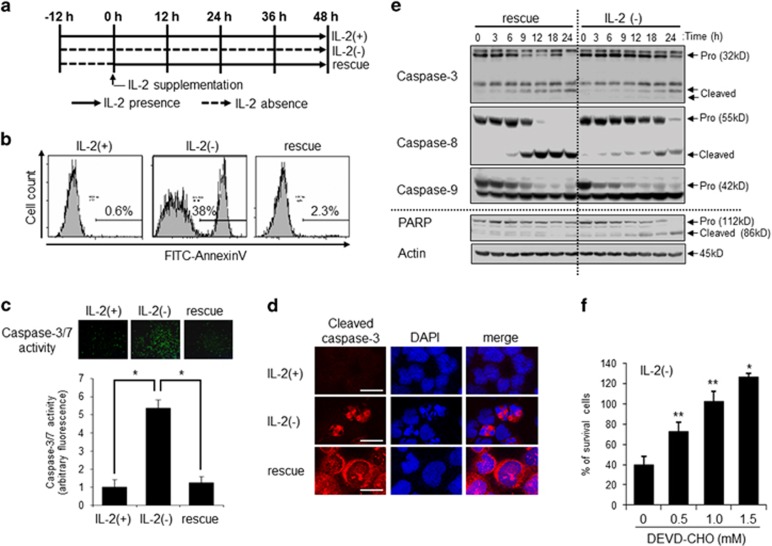
IL-2(−) induced caspase-dependent apoptosis. (**a**) Time schedule of study. KHYG-1 cells were cultured in RPMI1640 containing 10% FCS with (IL-2(+)) or without (IL-2(−)) IL-2 (100 U/ml). After 12 h of IL-2 deprivation, IL-2 (100 U/ml) was added to media (rescue), and this point was indicated as 0 h. (**b**) Cells were cultured at each condition for 24 h and stained with fluorescein isothiocyanate (FITC)–Annexin V. Percentage of FITC–Annexin V-positive cells were measured by flow cytometry. (**c**) *In vivo* caspase-3/-7 activity was performed with fluorescent substrates of caspase-3/7 (FAM-DEVD-FMK). Cells were cultured at each condition for 36 h. Images were obtained by fluorescent microscopy. The value is mean±S.D. from three different experiments. *P<0.005. (**d**) Cells were treated with IL-2(+), IL-2(−), and rescue for 12 h, and stained with anticleaved caspase-3 p17 antibody and DAPI. Images were obtained by confocal microscopy. Scale bar, 10 *μ*m. (**e**) The cells were incubated in IL-2(−) or rescue for the indicated times, and each protein was detected by immunoblotting. (**f**) Cells were incubated in IL-2(−) at each concentration of DEVD-CHO for 36 h. Surviving cells were counted with trypan blue and indicated as a percentage of 0 h. Values are mean±S.D. from three different experiments. *P<0.005 *vs* 0 h; **P<0.05 *vs* 0 h

We previously showed that IL-2(−) induced ceramide accumulation that mediated cell death with DNA condensation in KHYG-1 cells.^3^ The cells were incubated for 12 h in IL-2(−) and then untreated or IL-2 was added to the media (indicated as 0 h in Figure 1a). After 24 h of IL-2(−), apoptotic cells were detected with flow cytometry analysis of Annexin V, a marker of PS externalization and caspase-dependent apoptosis (Figure 1b). Annexin V-positive cells were increased in IL-2(−) compared with IL-2 containing medium (IL-2(+)). We confirmed our previous data that IL-2 supplementation (rescue) could prevent cells undergoing IL-2(−)-mediated apoptosis (Figure 1b). To examine caspase activation, we performed *in vivo* casapse-3/7 activity in KHYG-1 cells (Figure 1c). After 12 h, IL-2(−) induced activation of caspase-3/7, but IL-2 rescue inhibited its activation, suggesting IL-2(−) induced caspase-dependent apoptosis, and IL-2 rescue could rescue apoptosis. Pro-caspases are cleaved and the cleaved form activates apoptotic signal. As shown Figure 1d, cleaved caspase-3 was observed with nuclear condensation in some IL-2(−)-treated cells compared with IL-2(+)-treated cells. Interestingly, cleaved caspase-3 was also detected without nuclear condensation and localized in cytosol in rescued cells (Figure 1d). To confirm caspase cleavage in IL-2(−) and rescue cells, we performed western blotting analysis. Pro-caspase-3 (32 kD), pro-caspase-8 (55 kD), and pro-caspase-9 (42 kD) were cleaved by IL-2(−) treatment (Figure 1e). According to caspase cleavage, poly ADP ribose polymerase (PARP), a caspase-3 target and marker of apoptosis, was cleaved by IL-2(−) (Figure 1e). However, in rescue cells, PARP cleavage (86 kD) was not observed, even though caspases were cleaved (Figure 1e). These results suggested that IL-2(−) induced caspase activation and subsequent apoptosis, and rescue inhibited apoptosis through a mechanism involving inhibition of caspase activity but not caspase cleavage. Indeed, a caspase inhibitor, DEVD-CHO, inhibited IL-2(−)-induced cell death by a concentration-dependent mechanism (Figure 1f).

### Ceramide accumulated at lysosomes via ASM activation in IL-2(-)-treated cells

**Figure 2:**
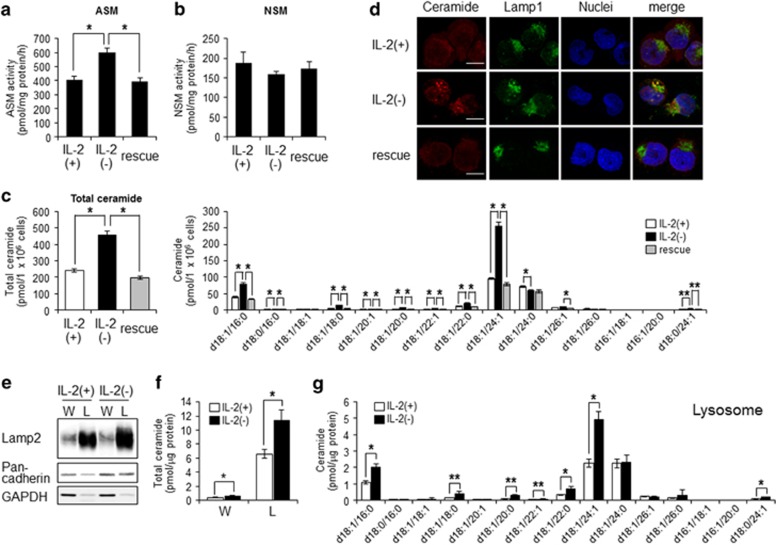
IL-2(−) induced accumulation of lysosomal ceramide. (**a** and **b**) KHYG-1 cells were cultured in IL-2(+), IL-2(−), and rescue conditions for 12 h. Cells were harvested, and after extraction of the proteins, the activities of each enzyme were assessed. Activities of ASM (**a**) and NSM (**b**) were measured using C_6_-NBD-ceramide and C_6_-NBD-SM as substrates. (**c**) After 36 h, ceramide levels were assessed by LC-MS/MS. (**d**) Cells were cultured at each condition for 12 h, fixed, stained with anti-ceramide and Lamp1 (lysosome) antibodies, and analyzed by confocal microscopy. Nuclei were counter-stained with DAPI. Scale bars, 10 *μ*m. (**e**) After 24 h, lysosome fraction were isolated as described in Materials and Methods section. Lamp2 (lysosome), pan-cadherin (plasma membrane), and GAPDH (cytosol) were used as markers for each organelle in whole cell lysate (W) and lysosome fraction (L). (**f** and **g**) Total ceramide levels in whole cell and lysosome (**f**) and ceramide species in lysosome (**g**) were assessed by LC-MS/MS. Values are mean±S.D. from three different experiments. *P<0.005, **P<0.05

Previously, we showed that ceramide generation in IL-2(−) is important for induction of apoptosis.^3^ We confirmed that IL-2(−) activated ASM but not neutral sphingomyelinase (NSM), enzymes that generate ceramide from sphingomyelin (Figures 2a and b). According to enhanced ASM activity, ceramide species such as d18:1/16:0, d18:1/18:0, and d18:1/24:1 were significantly increased by IL-2(−) (Figure 2c) but inhibited by IL-2 rescue (Figures 2a–c). ASM acts at the lysosome and outer leaflet of the plasma membrane after activation.^33^ Thus we assessed where ceramide was produced and accumulated by IL-2(−). As shown in Figure 2d, ceramide was increased and colocalized with Lamp1, a lysosomal marker, in IL-2(−) conditions. IL-2 rescue suppressed ceramide accumulation (Figure 2d). To elucidate accumulation of ceramide in lysosome, we performed lysosome fractionation after treatment with IL-2(+) or IL-2(−) (Figure 2e). As shown in Figures 2f and g, lysosomal ceramide was increased by IL-2(−). In addition, the species of increased ceramide (d18:1/16:0, d18:1/18:0, and d18:1/24:1) in lysosome were same as those in whole cells (Figures 2c and g). These data suggested that IL-2(−) induced lysosomal ceramide accumulation through activation of ASM and inhibition of SMS and glucosylceramide synthase and that lysosomal ceramide was related to IL-2(−)-induced apoptosis.

### IL-2(-) treatment induces XIAP degradation

**Figure 3:**
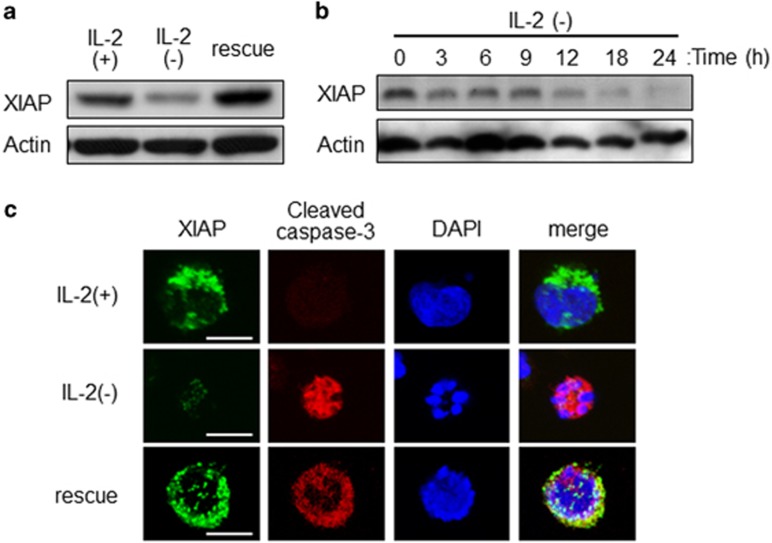
Involvement of XIAP degradation on caspase-dependent apoptosis during IL-2(−). (**a**) KHYG-1 cells were incubated in IL-2(+), IL-2(−), and rescue conditions for 12 h. XIAP and actin were detected by western blotting analysis. (**b**) Cells were harvested at the indicated times after IL-2(−) treatment. Immunoblotting was performed with anti-XIAP and anti-actin antibodies. (**c**) Cells were incubated at each condition for 12 h, fixed, stained with XIAP and cleaved caspase-3 antibodies, and analyzed by confocal microscopy. Scale bars, 10 *μ*m

To assess the mechanism that IL-2 rescue inhibits the activity of cleaved caspase, we focused on XIAP, an IAP with inhibitory effects on caspases.^11, 12^ XIAP inhibits both caspase-3 and caspase-9 through direct binding.^13, 14, 15, 16^ In the above data, cleavage of caspases was observed in IL-2 rescue without apoptosis. Thus we examined whether XIAP was related to IL-2(−) and rescue. XIAP proteins were decreased by IL-2(−) treatment and IL-2 rescue recovered XIAP levels (Figure 3a). IL-2(−) treatment promoted reduced XIAP levels from 3 h and after 24 h XIAP protein was not detectable (Figure 3b). To confirm XIAP degradation, we observed the localization of XIAP and cleaved caspase-3 by immunocytochemistry and confocal microscopy. As shown in Figure 3d, IL-2(-) treatment decreased XIAP, and cleaved caspase-3 accumulated in the nuclei. However, XIAP colocalized with cleaved caspase-3 in the cytosol by IL-2 rescue (Figure 3d). These data suggested IL-2(−) activates both capase-3 cleavage and XIAP degradation, and IL-2 rescue prevents XIAP degradation but not caspase-3 activation.

### CTSB inhibitor blocks XIAP degradation without ceramide accumulation in IL-2(−)

**Figure 4:**
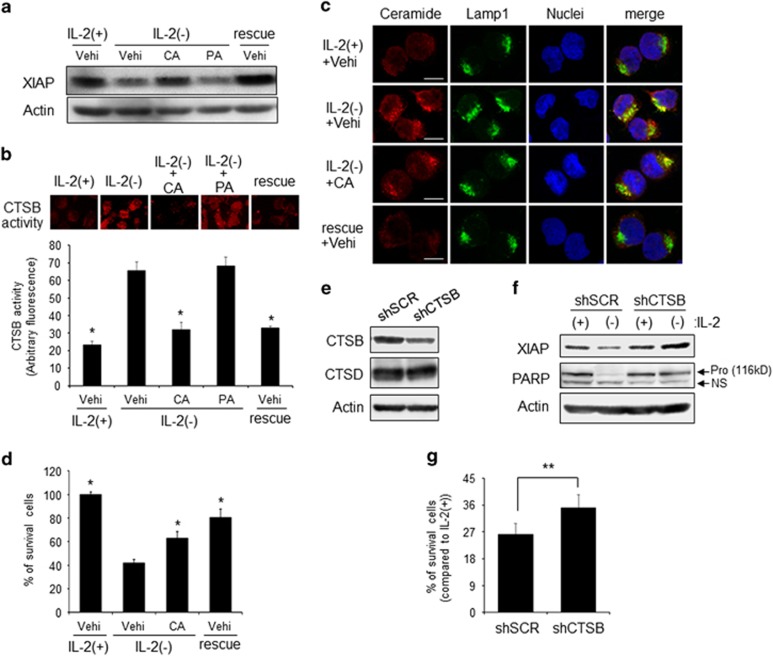
Involvement of CTSB in IL-2(−)-mediated apoptosis. KHYG-1 cells were treated with IL-2(−) with or without 4 *μ*M cathepsin inhibitors. (**a**) After 24 h of treatment, western blotting analysis was performed by using anti-XIAP antibody or anti-actin antibody. (**b**) After 12 h, *in vivo* CTSB activity was measured. Images were obtained by fluorescent microscopy. Values are mean±S.D. from three different experiments. *P<0.005 *vs* IL-2(−) with vehicle treatment. (**c**) After 12 h, cells were fixed and stained with anti-ceramide and anti-Lamp1 antibodies. Nuclei were counter-stained with DAPI. Images were obtained with confocal microscopy. Scale bars, 10 *μ*m. (**d**) After 24 h of treatment, viable cell numbers were counted by trypan blue staining and indicated as a percentage of IL-2(+). Values are mean±S.D. from three different experiments. *P<0.005 *vs* IL-2(−) with vehicle treatment. (**e**) Establishment of control and CTSB knockdown cells by using lentiviral particles of control shRNA (shSCR) and CTSB-specific shRNA (shCTSB). CTSB, CTSD, and actin were assessed by western blotting analysis. (**f**) After 24 h of treatment, each protein was detected by immunoblotting. (**g**) After 36 h, viable cell numbers were counted after trypan blue staining and indicated as a percentage of IL-2(+) in each cell. Values are mean±S.D. from four different experiments. **P<0.05

The above data suggested that lysosomal ceramide induced XIAP degradation and apoptosis during IL-2(−). Thus we examined whether the lysosomal apoptotic pathway is involved in IL-2(−)-induced apoptosis. Lysosomal protease CTSs such as CTSB and CTSD are well-known apoptosis inducers.^34^ Especially, CTSD is downstream of ceramide.^28, 29, 35^ To assess the relationship between the cathepsin pathway and IL-2(−)-induced XIAP degradation and apoptosis, we used cathepsin inhibitors. Pepstatin A, a CTSD inhibitor, had no effect on IL-2(−)-induced XIAP degradation (Figure 4a). Interestingly, a CTSB-specific inhibitor, CA-074 Me, prevented XIAP reduction similar to IL-2 rescue (Figure 4a). Next we demonstrated that IL-2-(−) activated CTSB *in vivo*, which was inhibited by CA-074 Me but not by pepstatin A treatment (Figure 4b).

To elucidate whether CTSB activation was downstream of increased lysosomal ceramide, we observed ceramide accumulation in IL-2(−) conditions with or without CA-074 Me treatment. As shown in Figure 4c, CA-074 Me did not prevent lysosomal ceramide accumulation. However, CA-074 Me treatment inhibited apoptosis (Figure 4d). To confirm the effects of CA-074 Me, we performed knockdown of CTSB. KHYG-1 cells were quite difficult to transduce genes transiently. Thus we established CTSB knockdown cell lines by using CTSB-specific shRNA lentivirus (Figure 4e). CTSB knockdown (shCTSB) blocked XIAP degradation and PARP cleavage induced by IL-2(−) (Figure 4f). In addition, cell survival was also increased in shCTSB cells compared with control cells (shSCR) (Figure 4f). These data suggested that accumulation of lysosomal ceramide during IL-2(−) activated CTSB but not CTSD and the subsequent apoptosis.

### ASM inhibitor suppressed ceramide accumulation and CTSB-mediated apoptosis

**Figure 5:**
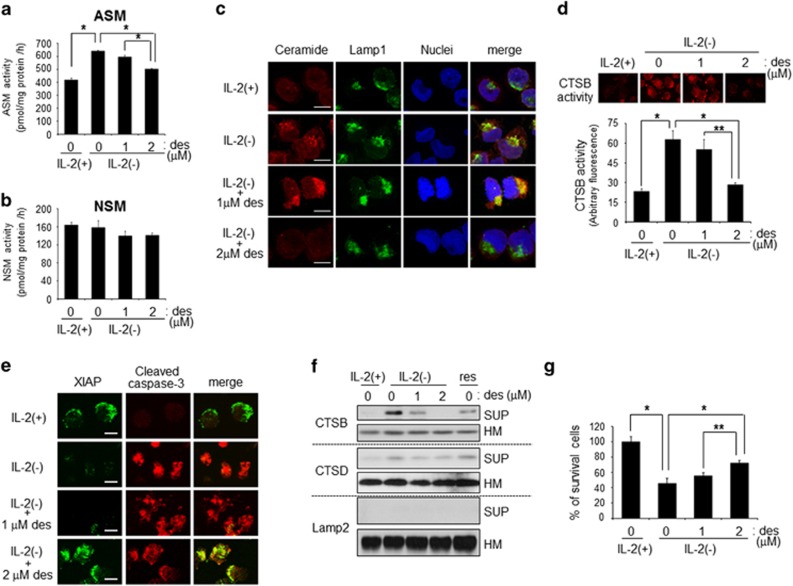
ASM inhibitor suppressed CTBS-mediated apoptosis. KHYG-1 cells were treated with IL-2(−) with or without ASM inhibitor, desipramine (des). (**a** and **b**) After 12 h, cells were harvested, and after extraction of the proteins, the activities of ASM (**a**) and NSM (**b**) were assessed using C_6_-NBD-SM as substrates. Values are mean±S.D. from three different experiments. *P<0.005. (**c**) After 12 h, cells were fixed and stained with anti-ceramide and anti-Lamp1 antibodies. Images were obtained with confocal microscopy. Scale bars, 10 *μ*m. (**d**) After 12 h, *in vivo* CTSB activity was performed. Images were obtained by using fluorescent microscopy. Values are mean±S.D. from three different experiments. *P<0.005, **P<0.05. (**e**) After 24 h, cells were harvested and homogenized. Endolysosomal (heavy membrane, HM) and cytosol (supernatant, SUP) fractions of homogenate were collected and analyzed by immunoblotting. Lamp2 was used as a heavy membrane (HM) marker. The results were the representative from three different experiments. (**f**) After 12 h, cells were stained with anti-XIAP and anticleaved caspase-3 antibodies. Images were obtained by confocal microscopy. Scale bars, 10 *μ*m. (**g**) After 24 h, viable cell numbers were counted after staining with trypan blue and indicated as a percentage of IL-2(+). Values were mean±S.D. from three different experiments. *P<0.005, **P<0.05

The above data showed that IL-2(−) activated ASM, which induced lysosomal ceramide accumulation. We tested the effects of an ASM inhibitor on IL-2(−)-mediated apoptosis. Preincubation with ASM inhibitor desipramine blocked ASM activation but not NSM during IL-2(−) (Figures 5a and b). Next, we observed IL-2(−)-induced lysosomal ceramide accumulation in the presence or absence of desipramine by immunocytochemistry. As shown in Figure 5c, ceramide accumulation induced by IL-2(−) was suppressed by desipramine treatment. Moreover, desipramine also blocked CTSB activation, which is downstream of lysosomal ceramide (Figure 5d).

CTSB or CTSD were released from lysosomes to the cytosol.^36, 37, 38^ Lysosomal ceramide activates CTSD through its release from lysosome membranes to the cytosol.^28^ To examine CTSB release by IL-2(−) and the effect of desipramine on CTSB release, we separated cytosol and heavy membrane fractions, which contain lysosome membranes. Interestingly, IL-2(−) induced CTSB release from lysosome to cytosol, and its release was inhibited by desipramine treatment (Figure 5e). According to inhibition of CTSB, XIAP degradation, which elicits cell survival, was also suppressed by desipramine treatment (Figure 5g).

IL-2(−) induced caspase-dependent apoptosis. Thus we assessed caspase-3 activation during desipramine treatment. Interestingly, desipramine inhibited XIAP degradation but had no effect on caspase-3 activation (Figure 5e). However, XIAP colocalized with cleaved caspase-3 during desipramine treatment. Desipramine treatment inhibited XIAP downregulation and suppressed apoptosis (Figure 5g).

**Figure 6:**
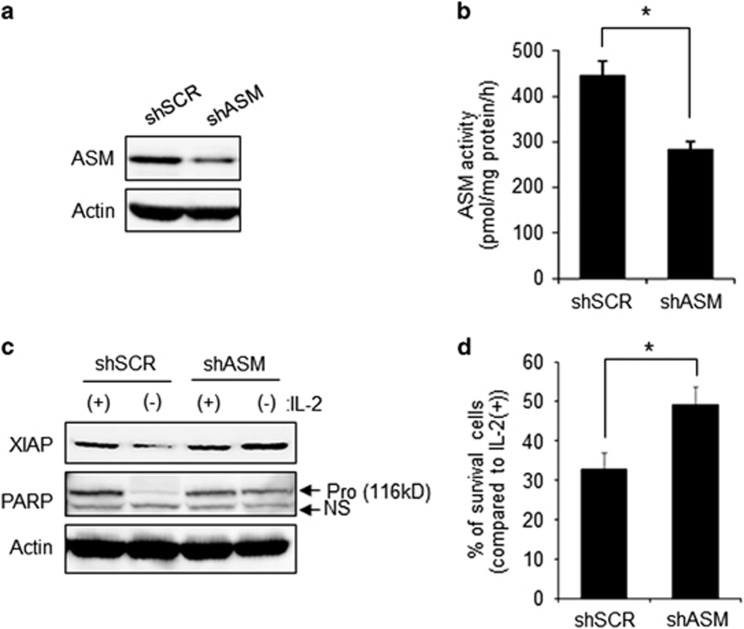
Knockdown of ASM inhibited IL-2(−)-mediated apoptosis. (**a**) Establishment of ASM knockdown KHYG-1 cells by using lentiviral particles of ASM-specific shRNA (shASM). Control cells (shSCR) were established as described in Figure 4e. ASM and actin were detected by western blotting analysis. (**b**) ASM activity was measured by using C_6_-NBD-SM as substrates. (**c**) After 24 h of treatment with IL−2(−) or IL-2(+), each protein was detected by immunoblotting. (**d**) After 36 h, viable cell numbers were counted after trypan blue staining and indicated as a percentage of IL-2(+) in each cell. Values are mean±S.D. from four different experiments. *P<0.005

To confirm the effect of desipramine treatment, we established ASM-knockdown cells by using ASM-specific shRNA lentivirus. As shown in Figures 6a and b, ASM protein and activity were significantly reduced by ASM-knockdown cells (shASM) compared with control cells (shSCR). According to knockdown of ASM, XIAP degradation and PARP cleavage by IL-2(−) were blocked in shASM cells (Figure 6c). In addition, cell survival was also improved by ASM knockdown (Figure 6d). These results suggested that IL-2(−)-activated ASM increased lysosomal ceramide, which activates CTSB via its release from the lysosome to cytosol for XIAP degradation.

### Exogenous ceramide mimics IL-2(-) to activate CTSB and mediate XIAP degradation

**Figure 7:**
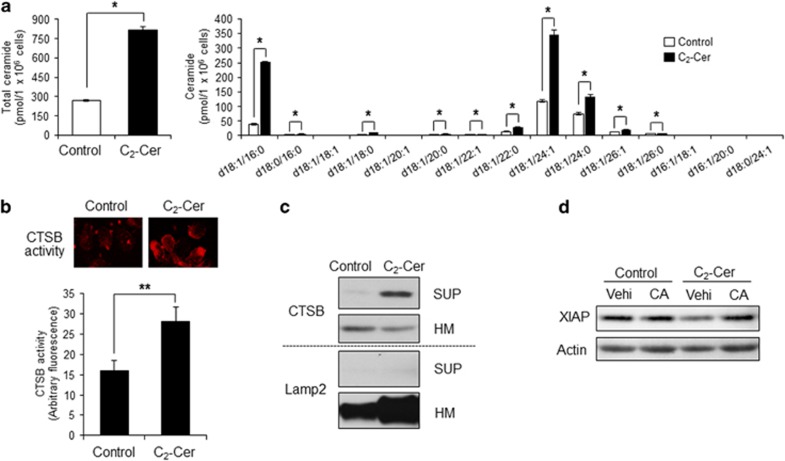
C_2_-ceramide induced activation and release of CTSB. (**a**) Cells were treated with 50 *μ*M C_2_-ceramide (C_2_-Cer) for 6 h. Ceramide amounts were measured by LC-MS/MS. Values are mean±S.D. from three different experiments. *P<0.005. (**b**) Cells were treated with 50 *μ*M C_2_-ceramide for 12 h. CTSB activity was measured by the Magic Red CTSB Activity Kit. Images were obtained by fluorescent microscopy. Values are mean±S.D. from three different experiments. *P<0.005, **P<0.05. (**c**) Release of CTSB was assessed by western blotting analysis after separating fractions of heavy membrane (HM) and cytosol (SUP). (**d**) XIAP levels were measured by immunoblotting using anti-XIAP antibodies. Results are representative of three independent experiments

From the above data, endogenous ceramide induced by IL-2(−) mediated CTSB activation, XIAP degradation, and caspase-dependent apoptosis. Previously, we have used permeable short chain *N*-acetyl sphingosine (C_2_-ceramide) to mimic the effects of endogenous ceramide on cell death, differentiation, and autophagy.^39, 40, 41^ Here, to examine whether exogenous C_2_-ceramide also induces ceramide-CTSB-mediated apoptosis, we treated KHYG-1 cells with C_2_-ceramide. As shown in Figure 7a, we measured ceramide amounts by liquid chromatography-electrospray ionization tandem mass spectrometry (LC-MS/MS) after treatment with C_2_-ceramide. Some ceramide species such as d18:1/16:0, d18:1/22:0, d18:1/24:1, and d18:1/24:0 were significantly elevated by C_2_-ceramide treatment and were same species induced by IL-2(−) treatment (Figures 2c and 7a). To elucidate whether exogenous ceramide also activates CTSB through its release from the lysosome to cytosol, we measured *in vivo* CTSB activity and examined the release of CTSB in C_2_-ceramide treatment (Figures 7b and c). After 12 h of treatment, C_2_-ceramide activated CTSB compared with vehicle treatment (control) (Figure 7b). Moreover, CTSB release from the lysosome to cytosol was also induced by C_2_-ceramide treatment (Figure 7c). Next, we assessed whether C_2_-ceramide-activated CTSB affected XIAP degradation. As shown in Figure 7d, C_2_-ceramide induced decrease of XIAP protein. Furthermore, CA-074 Me inhibited the degradation of XIAP induced by C_2_-ceramide (Figure 7d). These results suggested that exogenous ceramide also accelerates release and activation of CTSB with endogenous ceramide accumulation and mediates CTSB-dependent XIAP degradation.

### Exogenous ceramide induced XIAP degradation and caspase-dependent apoptosis

**Figure 8:**
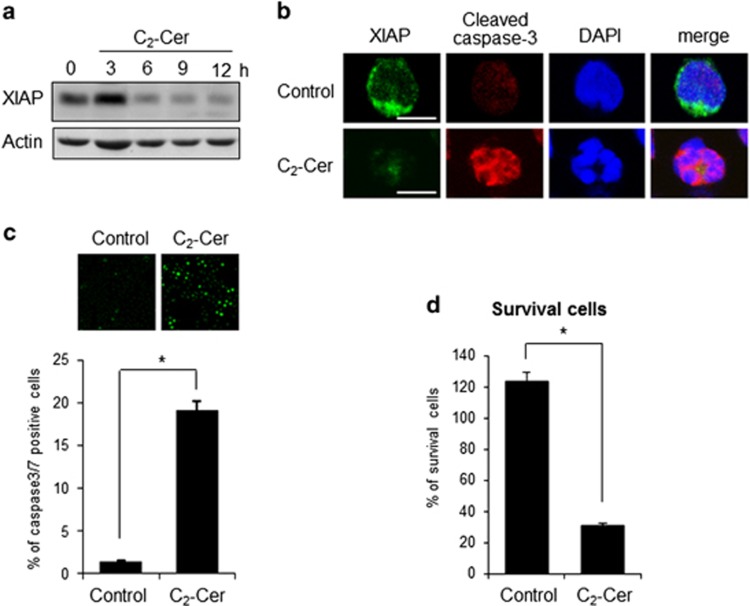
C_2_-ceramide induced apoptosis with XIAP degradation. (**a**) KHYG-1 cells were treated with 50 *μ*M C_2_-ceramide (C_2_-Cer) for the indicated times. XIAP protein was detected by western blotting analysis. (**b**) After 12 h of C_2_-Cer treatment, cells were stained with anti-XIAP and anticleaved caspase-3 antibodies. Nuclei were counterstained with DAPI. Images were obtained by confocal microscopy. Scale bars, 10 *μ*m. (**c**) *In vivo* caspase-3/-7 activity was measured by fluorescent substrate of caspase-3/7 (FAM-DEVD-FMK). Images were obtained by fluorescent microscopy. Values are mean±S.D. from three different experiments. *P<0.005. (**d**) After 12 h, viable cell numbers were counted after staining with trypan blue and indicated as a percentage of vehicle treatment. Values were mean±S.D. from three different experiments. *P<0.005

Finally, we examined the effect of exogenous ceramide on XIAP degradation and caspase-dependent apoptosis. C_2_-ceramide treatment reduced cytosolic XIAP protein by western blotting analysis and immunocytochemistry (Figures 8a and b). Active caspase-3 was detected by C_2_-ceramide treatment as demonstrated by XIAP degradation (Figure 8b). Moreover, C_2_-ceramide induced *in vivo* caspase-3 activation and apoptosis (Figures 8c and d). These data suggested that C_2_-ceramide mimicked IL-2(−)-induced apoptosis by XIAP degradation and caspase-3 activation.

## Discussion

KHYG-1 NK/T lymphoma cells require IL-2 for their proliferation and survival and undergo apoptosis by IL-2(−) even in the presence of serum. Thus it is important to understand the mechanism of IL-2(−)-induced apoptosis to develop novel treatments for drug-resistant NK/T cell lymphoma. We first showed that IL-2(−) induced caspase-dependent apoptosis, characterized by PS externalization and cleavage of caspase-8, -9, and -3, and the addition of IL-2 rescued cell death. Interestingly, although proteinase activity of caspase-3 was inhibited by IL-2 rescue, the 17–19-kD cleaved form of caspase-3 was observed. Furthermore, cleaved caspase-3 p17 did not traffic into the nucleus after IL-2 rescue while IL-2(−)-induced active caspase-3 accumulated in the nucleus to mediate the degradation of PARP. The precise mechanism of nuclear transport of caspase-3 has been clarified, but the translocation of caspase-3 p17 with more processing form p12 into the nucleus might be involved in Fas-induced apoptosis. Previous studies have demonstrated that p3-recognition site of caspase-3 is important for its nuclear entry.^42, 43^ Indeed, our data showed that more short form of cleaved caspase-3 (12 kD) was slightly but reliably detected in only IL-2(−), suggesting that caspase-3 was not cleaved completely in IL-2 rescue. In addition, caspase-3 p17 remained in the cytosol and did not move into the nucleus after rescue of apoptosis by IL-2 supplementation. These results suggested that the mechanism that inhibits the entry of caspase-3 p17 into the nucleus exists in the cytosol.

It is well known that ASM localizes in lysosomes. During IL-2(−) conditions, ASM-induced increase in ceramide was detected in lysosomes using anti-ceramide antibodies that colocalized with a lysosome marker Lamp1. After IL-2 rescue, the ceramide content in lysosomes decreased to the IL-2(+) control level. The molecular species of IL-2(−)-increased ceramide are of d18:1/16:0 and d18:1/24:1 types. We can detect that the increase of molecular species of ceramide by a diverse of stressors appears to be similar in pattern regardless of the mechanisms of PCD (data not shown). The localization of ceramide generated by the different pathways may be more critical for the regulation of cell death by ceramide signal, but at present evidence is ambiguous.

We detected *in vitro* activities of caspases in lysates of IL-2(−) and IL-2 rescued cells (data not shown). However, the *in vivo* activity of caspase-3 was significantly suppressed by IL-2 rescue. Because XIAP can directly bind to caspase-3 to inhibit cleaved activation,^14^ we focused on the regulation of XIAP protein, a member of the IAP family, by ASM-induced lysosomal ceramide.^14, 16^ A decrease of XIAP by IL-2(−) was restored by IL-2 rescue. This reduction was due to protein degradation but not the suppression of mRNA expression in XIAP (data not shown). It was reported that XIAP expression was controlled by activated NF-κB^44^ and that the PI3-K/Akt pathway, upstream of NF-*κ*B, regulated XIAP.^45^ We also detected NF-κB activation and enhanced XIAP mRNA expression by IL-2 rescue (data not shown). This suggests that the increase of XIAP in cell proliferation is regulated transcriptionally, whereas the decrease of XIAP induced by IL-2(−) is due to degradation at the protein level.

Why could not XIAP inhibit caspase cleavage? Recently, some reports showed that cleaved caspase has other effects such as inflammatory activation without apoptosis. Kavanagh *et al.*^46^ demonstrated that cytoplasmic cleaved caspase-3 induces pro-inflammatory activation through PKC*δ* but not apoptosis in microglia. Pro-inflammatory response activates NF-kB pathway. Moreover, Cheng *et al.*^47^ showed that ionizing radiation activates PKC*δ* through caspase-3/7 and leads production of growth factors via Akt pathway in pancreatic cancer cells. Indeed, rescue activated NF-*κ*B pathway and cell proliferation (data not shown). Thus cytoplasmic cleaved caspase might be related in cell proliferation but not apoptosis in rescue condition.

Recently, it was reported that the ceramide analogue LCL85 enhanced Fas-induced apoptosis by inhibiting XIAP.^48^ We here demonstrated that regulation of ceramide in lysosomes has a role in the degradation of XIAP. What is the mechanism by which XIAP is degraded by lysosomal ceramide? Mechanisms that decrease XIAP protein include degradation by ubiquitination and proteases and/or transcriptional regulation.^49^ Ceramide mediates apoptosis, and many molecules, including ceramide-activated protein phosphatases and protein kinase C *ζ*, are involved in its signaling.^50, 51^ Especially, lysosomal aspartic protease CTSD is reported to be activated by ASM-generated ceramide to induce apoptotic cell death in response to TNF signaling.^28, 35^ Lysosomal ceramide generated by ASM bound to CTSD mediated its translocation to the cytosol, following activation of the mitochondrial apoptotic pathway. ^28^ Interestingly, we found that an inhibitor of cysteine protease CTSB (CA-074 Me), but not CTSD (pepstatin A), inhibited XIAP degradation, and activation of CTSB was detected after IL-2(−) treatment. Inhibitors of CTSB suppressed IL-2(−)-mediated apoptotic cell death, suggesting a role for CTSB, but not CTSD, in apoptosis. However, previous studies indicated a role for CTSB in tumor progression.^52^ The increased expression of CTSB was related to the invasion and proliferation of melanoma cells,^9^ and CTSB decreased dramatically after serum-deprivation-induced apoptosis, whereas CTSD increased, suggesting a balance between CTSB and CTSD.^53^ In the current study, we showed that lysosomal ceramide-activated CTSB induced caspase activation through XIAP degradation because an inhibitor of CTSB CA-074 Me was effective in blocking the apoptotic pathway. However, we could not detect the cleavage site of CTSB in XIAP protein or direct digestion of XIAP using cell lysates and recombinant CTSB (data not shown). Bien *et al.*^54^ showed that doxorubicin activated CTSB and CTSB-mediated caspase-dependent apoptosis in HeLa cells. In addition, the downregulation of XIAP was also induced by doxorubicin and was inhibited by CTSB siRNA or CA-074 Me treatment. These data support our finding that CTSB regulates XIAP degradation.

CA-074 Me did not inhibit the increase of ceramide in lysosomes during IL-2(−), suggesting that ASM-induced ceramide generation is upstream of CTSB activation. Although CTSB has not been reported to bind to ceramide, previous studies showed that CTSB release is induced by alterations of lysosomal membrane permeabilization (LMP) in response to various stimuli, which activates caspase-mediated apoptosis.^55^ Thus lysosomal ceramide might induce LMP to release CTSB to the cytosol, but the precise mechanism of CTSB activation by lysosomal ceramide is unknown.

ASM activation and ceramide generation have been reported in various signaling pathways triggered by numerous stimuli,^25, 56^ chemotherapeutic drugs,^30, 57^ irradiation,^58, 59^ or pathogens.^27, 60^ In the present study, we showed IL-2(−) activated ASM, but not NSM, and induced ceramide accumulation in lysosomes, suggesting that lysosomal ceramide is critical for IL-2(−)-induced apoptosis. Indeed, an inhibitor of ASM, desipramine, inhibited IL-2(−)-induced apoptosis by suppressing ceramide generation in lysosomes. Desipramine also inhibited the nuclear activation of caspase-3 through the degradation of XIAP by CTSB activation.

Finally, we investigated whether exogenous C_2_-ceramide mimics IL-2(−)-mediated CTSB activation, XIAP degradation, and caspase-dependent NK/T lymphoma cell apoptosis. C_2_-ceramide increased apoptosis by generating physiological d18:1/16:0 and d18:1/24:1 ceramides similar to IL-2 deprivation. The increase of ceramides induced activation of CTSB and subsequent XIAP degradation. Huang *et al.*^61^ described that exogenous treatment with C_2_-ceramide triggered lysosomal pathways in T cell hybridomas and A549 lung adenocarcinoma cells and that C_2_-ceramide might have no direct effect on lysosomal function because the inhibition of ASM did not affect LMP or apoptosis. Recently, sphingosine, a metabolite of ceramide via ceramidase, was reported to mediate LMP and relocation of CTSB in TNF-*α*-treated hepatoma cells.^55^ Thus sphingosine generated by acid ceramidase from ASM-generated ceramide might activate CTSB during IL-2(−). In fact, IL-2(−) also increased sphingosine (18 : 1) (from 3.35±0.85 pmol/1 × 10^6^ cells in IL-2(+) to 4.97±0.27 pmol/1 × 10^6^ cells in IL-2(−)) (data not shown). However, amount of ceramide (459.3±21.4 pmol/1 × 10^6^ cells in IL-2(−)) was more than 100-fold of sphingosine. In addition, we investigated the effects of inhibitors of enzymes related in ceramide metabolism on C_2_-ceramide-induced cell death (Supplementary Figure S1). At first, the treatment of desipramine with C_2_-ceramide had no effect on cell survival. This result suggested that ASM is not involved in cellular metabolism of C_2_-ceramide in our case. Next, we used inhibitors of ceramide synthase and acid ceramidase, which inhibit the metabolism of ceramide from sphingosine and of sphingosine from ceramide, respectively. Fumonisin B1, which is ceramide synthase inhibitor to block ceramide production from sphingosine, recovered cell survival compared with C_2_-ceramide only. Inversely, acid ceramidase inhibitor D-NMAPPD enhanced C_2_-ceramide-medaited cell death. Acid ceramidase produces sphingosine from ceramide in lysosome. These results demonstrated that ceramide but not sphingosine is related in C_2_-ceramide-induced apoptosis. In addition, some reports that acid ceramidase is implicated in cell survival of malignant cells supported our data that acid ceramidase has inhibitory effect on C_2_-ceramide-mediated apoptosis.^62, 63, 64^ Thus we believe ASM-generated ceramide affected IL-2(−)-induced apoptosis.

In summary, our present work demonstrated a novel pathway related to lysosomal ceramide, CTSB, and XIAP in IL-2(−)-induced NK/T lymphoma cell apoptosis. Lysosomal ceramide generated by ASM induced the release of CTSB into the cytosol and the degradation of XIAP, resulting in the nuclear entry of active caspase-3 and subsequent apoptosis. The pathophysiological implication of this lysosomal ceramide/CTSB/XIAP axis in apoptotic cell death should be clarified in the future to develop a targeting therapy for NK/T lymphoma.

## Materials and Methods

### Materials

Materials were purchased as follows: human recombinant IL-2 (Imunase 35) (Shionogi Pharmaceutical, Osaka, Japan); C_6_-NBD ceramide, C_6_-NBD sphingomyelin, and C_2_-ceramide (Matreya, Pleasant Gap, PA, USA); CTSB specific inhibitor IV (CA-074 Me) (Merck Millipore, Darmstadt, Germany); pepstatin A and desipramine (Sigma-Aldrich, St. Louis, MO, USA); anti-caspase-8, anti-caspase-9, anti-PARP, antilysosomal-associated membrane protein 1 (Lamp1), anti-Lamp2, anti-ASM, anti-CTSB, anti-CTSD, and anti-*β*-actin antibodies (Santa Cruz Biotechnology Inc., Santa Cruz, CA, USA); Annexin V-conjugated FITC and monoclonal antibody against XIAP (BD Biosciences, Beverly, MA, USA); antiactive caspase-3 (p17) antibody and horseradish peroxidase–conjugated secondary antibodies (Promega, Madison, WI, USA); anti-caspase-3, anti-pan-cadherin, and anti-glyceraldehyde 3-phosphate dehydrogenase (GAPDH) antibodies (Cell signaling, Danvers, MA, USA); and anti-ceramide monoclonal IgM Ab clone (NHCER-2) was produced previously in our lab.^65^ Alexa Fluor-conjugated (AF-conjugated) secondary antibodies were from Molecular Probes (Eugene, OR, USA). 4',6-Diamidino-2-phenylindole (DAPI) was obtained from Roche Applied Science (Penzberg, Germany).

### Cell culture and counting of viable cells

IL-2 deprivation (IL-2(−)) and re-supplementation of human KHYG-1 cells were described previously (Figure 1a).(3) Cells were grown in RPMI 1640 (Sigma) supplemented with 10% fetal bovine serum and IL-2 (100 mU/ml) (IL-2–containing medium, IL-2(+)) at 37 °C in a humidified atmosphere containing 5% CO_2_. For IL-2 rescue experiments, cells were cultured in IL-2-depleted medium for 12 hours. Then, IL-2 (100 mU/ml) was supplied to media, and cells were kept thereafter (rescue). IL-2(−) indicated that cells were cultured in IL-2-deprived medium. Viable cell numbers were counted by 0.25% (w/v) trypan blue dye exclusion method at the indicated period.

For establishments of ASM or CTSB knockdown cells, lentiviral particles of shRNA were obtained from Santa Cruz. Cells were infected with control shRNA (shSCR, sc-108080), ASM-specific shRNA (shASM, sc-41650-v), and CTSB-specific shRNA (shCTSB, sc-29238-v) lentiviral particles after the treatment with 1 *μ*g/ml polybrene. Then cells were treated with 2 *μ*g/ml puromycin for establishment of knockdown cell lines. Efficiency of knockdown was checked by western blotting analysis.

### Detection of apoptotic cells by flow cytometry

Apoptotic cells were detected by PS exposure by staining with Annexin V-FITC and analyzed by flow cytometry. Cells were washed twice with ice-cold phosphate-buffered saline (PBS) and suspended in Annexin V binding buffer (BD Biosciences). After 15 min of staining with Annexin V–FITC and 7AAD, apoptotic cells were analyzed by flow cytometry (Gallios, Beckman Coulter, Miami, FL, USA) according to the manufacturer's protocol. For each group, a minimum of 10 000 cells were collected.

### Analysis of *in vivo* caspase and CTSB activities

*In vivo* caspase-3/7 activity was measured by FAM-fluorochrome inhibitor of caspases (FLICA) Caspase 3&7 Assay Kit (Immunochemistry Technologies, Bloomington, MN, USA) according to the manufacturer's information. Briefly, cells were incubated with the FAM-DEVD-fluoromethylketone (FMK) reagent for 1 h at 37 °C. After washing with PBS, cells were fixed with 0.1% (w/v) formaldehyde solution. *In vivo* CTSB activity was performed using Magic Red CTSB Activity Kit (Immunochemistry Technologies) according to the manufacturer's protocol. Cells were incubated with Magic Red CTSB Substrate (Immunochemistry Technologies) for 1 h at 37 °C. The cells were pelleted after centrifugation, washed with PBS, and fixed with 0.1% formaldehyde. Fluorescence was measured by a fluorescent microplate reader Infinite 500 (Tecan Group Ltd., Männedorf, Switzerland), or at least 200 cells were counted and indicated as the percentage of positive cells per total cells. Images were obtained by fluorescent microscopy Leica DMRB or confocal microscopy Leica TSC SP2 (Leica Microsystems, Wetzlar, Germany).

### Western blotting analysis

KHYG-1 cells were harvested, washed with PBS, and lysed in lysis buffer (10 mM Tris-HCl, pH 7.4, 10 mM KCl, 1.5 mM MgCl_2_, 1% (w/v) Triton X-100, 1 mM PMSF, 10 μg/ml leupeptin, and 10 *μ*g/ml aprotinin). After incubation on ice for 20 min, debris was removed by centrifugation at 2000 × *g* for 10 min at 4 °C. Supernatant was used as a loading sample. Proteins (30 *μ*g) were subjected to SDS-poly-acrylamide gel electrophoresis and transferred to polyvinylidene difluoride membranes (Millipore, Bedford, MA, USA). Nonspecific binding was blocked by incubation of the membrane with PBS containing 0.1% (w/v) Tween-20 (PBS-T) and 5% (w/v) non-fat dried milk for 20 min at room temperature. Then membrane was incubated with primary antibodies overnight at 4 °C and with secondary antibodies for 45 min at room temperature. Immunoreactive protein bands were visualized using an ECL-peroxidase detection system (Amersham Biosciences, Piscataway, NJ, USA) and LAS-4000 (Fujifilm, Tokyo, Japan).

### Acid and NSM activities

KHYG-1 cells were sonicated in lysis buffer (10 mM Tris-HCl, pH 7.5, 1 mM EDTA, 0.1% (w/v) Triton X-100, 1 mM phenylmethylsulfonyl fluoride (PMSF), 2.5 *μ*g/ml leupeptin, and 2.5 *μ*g/ml aprotinin) and incubated on ice for 30 minutes. The homogenate was centrifuged at 10 000 × *g* for 10 min at 4 °C, and the supernatant was used as an enzyme source. Proteins (100 μg) were mixed in reaction buffer for magnesium-dependent NSM (0.1 M Tris-HCl, pH 7.5, 10 *μ*M C_6_-NBD sphingomyelin, 10 mM MgCl_2_, 0.1% (w/v) Triton X-100, 5 mM dithiothreitol) or for ASM (0.1 M sodium acetate (pH 5.0), 10 *μ*M C_6_-NBD sphingomyelin, 0.1% (w/v) Triton X-100) and incubated for 90 min at 37 °C. Lipids were extracted using chroloform/methanol, applied to thin layer chromatography plates, and separated in chloroform/methanol/12 mM MgCl_2_ in D.W. (65 : 25 : 4, v/v/v). Fluorescent lipids were detected using LAS-4000 (Fujifilm) and quantified using MultiGauge 3.1 (Fujifilm).

### Ceramide measurement by LC-MS/MS

Lipid extraction from KHYG-1 cells (1 × 10^6^ cells) and measurements of ceramide species in lipid extracts were performed using LC-MS/MS as described previously.^66^ The amounts of ceramide species with various carbon chains (d18:1/16:0, d18:0/16:0, d18:1/18:1, d18:1/18:0, d18:1/20:1, d18:1/20:0, d18:1/22:1, d18:1/22:0, d18:1/24:1, d18:1/24:0, d18:1/26:0, d16:1/18:1, d16:1/20:0, and d18:0/24:1) were determined.

### Immunocytochemistry

For detection of active caspase-3 and XIAP, cells were treated by the indicated conditions, washed with ice-cold PBS, cytospun onto slides, and then fixed with 2% formaldehyde for 15 min at 4 °C. Fixed cells were permeabilized with PBS containing 0.1% Triton X-100 for 5 min and incubated with PBS containing 2% (w/v) BSA for 30 min at room temperature. Cells were washed with PBS and then incubated with primary antibodies for 90 min at room temperature. After washing with PBS, AF488- or AF546-conjugated anti-IgG antibodies were incubated for 45 min. For determination of lysosomal ceramide, fixed cells were treated with anti-ceramide antibody and anti-Lamp1 antibody for 90 min at room temperature. Then AF488-conjugated anti-IgM and AF546-conjugated anti-IgG antibodies were incubated for 45 min at room temperature. Nuclei were counterstained with DAPI.

### Cell fractionation

Lysosome fraction was collected by using the Lysosome Enrichment Kit for Tissue and Cultured Cells according to the manufacturer's protocol (Thermo Scientific, 89839, Rockford, IL, USA). Cells (1 × 10^7^ cells) were treated with IL-2(+) or IL-2(−) for 24 h, and cell pellets were used for lysosome isolation. Whole cell lysate before lysosome collection and isolated lysosome fractions were then subjected to western blotting analysis to check the purity of the isolated lysosome and measurement of SM by LC-MS/MS. Anti-Lamp2 (lysosome), anti-pan-cadherin (plasma membrane), and anti-GAPDH (cytosol) antibodies were used as markers of each organelle.

### CTSB and CTSD release

The release of CTSB and CTSD from the endolysosomal compartment was analyzed by heavy membrane fractionation.^67^ Cells were lysed in homogenization buffer (10 mM Hepes-KOH, pH 7.4, 1 mM EDTA, 0.25 M sucrose, 1 mM PMSF, inhibitor cocktail (Roche)), incubated on ice for 40 min, and homogenized by 27-G syringes. Homogenates were centrifuged at 300 × *g* for 5 min, and unbroken cells were removed. Then lysate was centrifuged at 20 000 × *g* for 30 min, and supernatants contained the cytoplasmic fraction. Pellets were resolved in homogenization buffer containing 1% Triton X-100 and used for heavy membrane fraction, including lysosomes. Proteins (40 *μ*g) were used for western blotting to detect the release of CTSB and CTSD from the heavy membrane fraction to the cytosol fraction. Anti-Lamp2 antibody was used as a lysosomal marker.

### Statistical analysis

Comparisons between two groups were carried out using the unpaired Student's *t*-test.

## Abbreviations

ASM: acid sphingomyelinase
CTSB: cathepsin B
CTSD: cathepsin D
GAPDH: glyceraldehyde 3-phosphate dehydrogenase
IL-2: interleukin-2
LC-MS/MS: liquid chromatography-electrospray ionization tandem mass spectrometry
NK/T: natural killer T
NSM: neutral sphingomyelinase
PS: phosphatidylserine
shRNA: short hairpin RNA
SMS: sphingomyelin synthase
XIAP: X-linked inhibitor of apoptosis protein
