# Supplementary material for: Lysosomal ceramide generated by acid sphingomyelinase triggers cytosolic cathepsin B-mediated degradation of X-linked inhibitor of apoptosis protein in natural killer/T lymphoma cell apoptosis
Source: Cell Death Dis. 2015 Apr 9;6(4):e1717–. doi: 10.1038/cddis.2015.82 (PMC4650549; doi:10.1038/cddis.2015.82)
Supplement: Supplementary Information [file cddis201582x1.docx]

**Supplementary Information**

**Materials and Methods**

***Materials***- Materials were purchased as follows: Fumonisin B1 (Merch Millipore, Darmstadt, Germany); D-NMAPPD (Santa Cruz Biotech).

**Supplementary Figure legends**

**Supplementary Figure S1. Effects of sphingolipid metabolism inhibitors on C_2_-ceramide-mediated apoptosis in KHYG-1 cells**

KHYG-1 cells (3 x 10^5^ cells) were treated with 50 μM C_2_-ceramide with or without inhibitors such as desipramine (ASM inhibitor), fumonisin B1 (FB1, CerS inhibitor), and D-NMAPPD (acid ceramidase inhibitor). After 6 hour, survival cells were detected with cell counting kit-8 and micro platereader (OD_450_). Values were means ± S.D. from four experiments. **P* < 0.005.
