# Supplementary figures and images for: Lysosomal ceramide generated by acid sphingomyelinase triggers cytosolic cathepsin B-mediated degradation of X-linked inhibitor of apoptosis protein in natural killer/T lymphoma cell apoptosis
Source: Cell Death Dis. 2015 Apr 9;6(4):e1717–. doi: 10.1038/cddis.2015.82 (PMC4650549; doi:10.1038/cddis.2015.82)

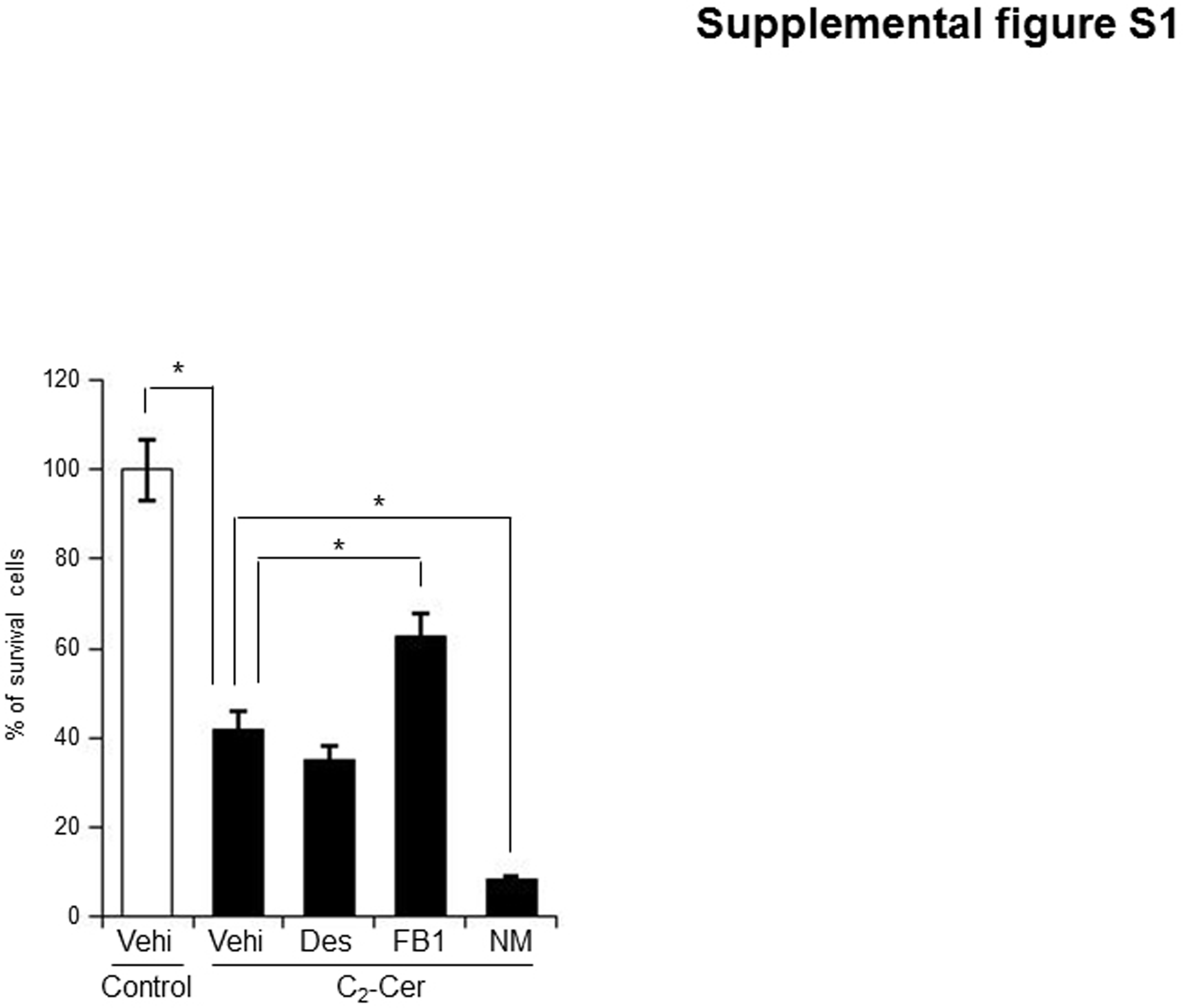

Supplement: Supplementary Figure S1 [file cddis201582x2.tif]
